# Genomic Markers Reveal Introgressive Hybridization in the Indo-West Pacific Mangroves: A Case Study

**DOI:** 10.1371/journal.pone.0019671

**Published:** 2011-05-11

**Authors:** Mei Sun, Eugenia Y. Y. Lo

**Affiliations:** 1 Department of Organismic and Evolutionary Biology, Harvard University, Cambridge, Massachusetts, United States of America; 2 School of Biological Sciences, The University of Hong Kong, Hong Kong, People's Republic of China; 3 Department of Ecology and Evolutionary Biology, Yale University, New Haven, Connecticut, United States of America; East Carolina University, United States of America

## Abstract

Biodiversity of mangrove ecosystems is difficult to assess, at least partly due to lack of genetic verification of morphology-based documentation of species. Natural hybridization, on the one hand, plays an important role in evolution as a source of novel gene combinations and a mechanism of speciation. However, on the other hand, recurrent introgression allows gene flow between species and could reverse the process of genetic differentiation among populations required for speciation. To understand the dynamic evolutionary consequences of hybridization, this study examines genomic structure of hybrids and parental species at the population level. In the Indo-West Pacific, *Bruguiera* is one of the dominant mangrove genera and species ranges overlap extensively with one another. Morphological intermediates between sympatric *Bruguiera gymnorrhiza* and *Bruguiera sexangula* have been reported as a variety of *B. sexangula* or a new hybrid species, *B. × rhynchopetala*. However, the direction of hybridization and extent of introgression are unclear. A large number of species-specific inter-simple sequence repeat (ISSR) markers were found in *B. gymnorrhiza* and *B. sexangula*, and the additive ISSR profiling of *B. × rhynchopetala* ascertained its hybrid status and identified its parental origin. The varying degree of scatterness among hybrid individuals in Principal Coordinate Analysis and results from NewHybrids analysis indicate that *B. × rhynchopetala* comprises different generations of introgressants in addition to *F*_1_s. High genetic relatedness between *B. × rhynchopetala* and *B. gymnorrhiza* based on nuclear and chloroplast sequences suggests preferential hybrid backcrosses to *B. gymnorrhiza*. We conclude that *B. × rhynchopetala* has not evolved into an incipient hybrid species, and its persistence can be explained by recurrent hybridization and introgression. Genomic data provide insights into the hybridization dynamics of mangrove plants. Such information can assist in biodiversity assessment by helping detect novel taxa and/or define species boundaries.

## Introduction

Mangrove forests consist of an important group of woody plants occupying coastal zone habitats. Global distributions of mangroves are mainly influenced by temperature [1], restricting them to warm tropical and subtropical latitudes in the Indo West Pacific (IWP) and Atlantic East Pacific (AEP) regions. Although these plants and associated organisms and habitats constitute one of the world's most productive ecosystems [2], extant mangrove taxa worldwide remain incompletely described and poorly identified, which limits our understanding of mangrove biodiversity and evolutionary relationships among the major constituents. In addition to convergent evolution in morphology, frequent appearance of new taxonomic entities through natural hybridization may have contributed to the difficulty in mangrove biodiversity assessment.

Natural hybridization is common in plants and plays a very important role in evolution as a source of novel gene combinations and a mechanism of speciation [3]–[10]. However, recurrent hybridization and introgression result in gene flow between species which could reverse the process of genetic differentiation among populations required for speciation. To understand the dynamic evolutionary consequences of hybridization, more genetic studies are needed to compare the hybrids and their parental species at the population and genomic level. Such studies are also important for accurately classifying and managing biodiversity.

Conventional approaches to hybrid identification are primarily based on their morphological intermediacy between parental species. However, many morphological characters are under environmental influences and some true hybrids may not always display an intermediate phenotype if the diagnostic traits are not controlled by codominant genes or genes of additive effects [11]. The inadequacy of morphological approach alone in studying natural hybridization is well recognized [12 and references therein]. More recent studies have employed various molecular technologies for identifying natural hybrids, for detecting introgression, and for studying hybrid speciation [e.g., 10–14].

*Bruguiera* Savigny is one of major mangrove genera of Rhizophoraceae. The genus consists of only six species which are divided into two groups according to flower size and pollinator type [1]. The two large-flowered species, *Bruguiera gymnorrhiza* (L.) Lamk. and *Bruguiera sexangula* (Lour.) Poir., have wide distributions in the Indo-West Pacific region and frequently occur in sympatry throughout Southeast Asia and tropical Australia. Morphological similarities between the two species have often led to identification uncertainties [15], and the presence of intermediate forms in sympatric populations further contributes to taxonomic difficulties. Ko (1978) treated the intermediate forms occurring in China as a variant of *B. sexangula*, and named it *Bruguiera sexangula* var. *rhynchopetala* Ko [16]. The observations of several morphological attributes that are intermediate between *B. gymnorrhiza* and *B. sexangula* suggest that this new taxon is most likely an interspecific hybrid, and a hybrid species name, *Bruguiera* × *rhynchopetala* (Ko) X. J. Ge et N. C. Duke, was thus proposed to denote its putative hybrid origin [17]. Based also on morphological observations, hybridization and introgression between *B. gymnorrhiza* and *B. sexangula* in Sri Lanka was speculated but yet to be confirmed [18]. In addition, the frequency of hybridization and extent of introgression in different geographical locations is unknown for the genus.

Various molecular markers have been explored for genetic investigation of plant hybridization. Of which, a large number of inter-simple sequence repeat (ISSR) markers can be easily generated across the genome for comparative analysis of the putative hybrids and their parental species. Wolfe et al. (1998) have shown that ISSR markers could offer a high degree of resolution to relationships and patterns of introgression than other types of molecular data used in hybridization studies [11]. In this study, we investigate natural hybridization in *Bruguiera* over a wide geographical area in the IWP region using ISSR together with sequence markers. Specifically, we address the following questions: (1) Do molecular data support the morphology-based field identification of *Bruguiera* × *rhynchopetala*? (2) Are *B. gymnorrhiza* and *B. sexangula* the only parental species involved in hybridization where other congeners also occur in sympatry? (3) What is the direction and extent of introgression at each of the examined geographical locations? (4) Has *Bruguiera* × *rhynchopetala* genetically differentiated from the parental species to deserve a separate hybrid species status?

## Materials and Methods

### Plant sampling

Fresh young leaves were collected from individual trees in four mangrove forests, including one site in Hainan Island of South China Sea, one site in North Sulawesi of Indonesia, and two sites in northeastern Australia. The sampled trees were targeted to include diverse morphs of parental species and their putative hybrids present at each site (Table 1). Hybrids were identified based on their unique intermediacy or a combination of morphological characteristics of the putative parents. All individual samples were assigned a field collection identification number and kept separately for genetic analyses.

**Table 1:** Geographical location, sample size, and number of ISSR fragments detected in *Bruguiera gymnorrhiza*, *B. sexangula*, *B. cylindrica*, *B. parviflora*, and the putative hybrid *B*. × *rhynchopetala*.

Sample site	Geographical coordinates	Taxon	Label	No. of individuals	No. of ISSR fragments
Australia					
Embley River, Weipa, Queensland	12°43′S, 142°02′E	*B. gymnorrhiza*	BG…ER	15 (16)	112
		*B. sexangula*	BS…ER	6 (6)	107
		*B*. × *rhynchopetala*	BR…ER	4 (4)	144
		*B. cylindrica*	BC…ER	8 (4)	104
		*B. parviflora*	BP…ER	2 (2)	70
Johnstone River, Queensland	17°30′S, 146°04′E	*B. gymnorrhiza*	BG…JR	2 (2)	124
		*B. sexangula*	BS…JR	1 (2)	79
		*B*. × *rhynchopetala*	BR…JR	1 (1)	88
**Hainan Island, China**					
Dongzhai Mangrove Nature Reserve	20°00′N, 110°35′E	*B. gymnorrhiza*	BG…HN	17 (4)	157
		*B. sexangula*	BS…HN	17 (4)	130
		*B*. × *rhynchopetala*	BR…HN	18 (2)	165
**Indonesia**					
North Sulawesi	1°22′N, 124°33′E	*B. gymnorrhiza*	BG…In	11 (6)	125
		*B. sexangula*	BS…In	7 (4)	109
		*B*. × *rhynchopetala*	BR…In	3 (3)	121

Numbers in parentheses indicate number of individuals included in ITS and chloroplast sequence amplification.

The sample size varied among sites depending on availability of identifiable hybrids in each mangrove forest. On the northeastern coast of Hainan Island, *B. gymnorrhiza* and *B. sexangula* partly overlap in Dongzhai Mangrove Nature Reserve, and frequent occurrence of intermediate forms at this site resulted in a discernible hybrid zone. In contrast, the hybrid forms were relatively rare or difficult to ascertain in the Indonesian population located in North Sulawesi, as in the two coastal river populations in northeastern Australia, the Embley River (ER) and the Johnstone River (JR) of Queensland. In addition to *B. gymnorrhiza* and *B. sexangula*, two other congeneric species, *Bruguiera cylindrica* (L.) Bl. and *Bruguiera parviflora* (Roxb.) Wight & Arn. ex Griff., coexist in the Embley River mangrove forest. Samples of all four *Bruguiera* species and putative hybrids were taken from this site for comparative analysis to determine parentage involved in hybridization.

### DNA extraction and ISSR amplification

Fresh young leaves were taken and stored individually at 4°C or dried and preserved with silica gel in plastic bags until DNA extraction. All dry leaf samples were kept in an electronic auto-dry cabinet (WEIFO, Taiwan). Total genomic DNA of each individual sample was extracted using a modified method of [19] for fresh leaves, and DNeasy Plant Mini Kit (QIAGEN) was used for silica gel-dried leaves to improve DNA yield and quality.

A large number of ISSR primers of Set No. 9 (Biotechnology Laboratory, University of British Columbia) were initially tested for PCR amplification. Twelve of the tested primers were selected for use based on the repeatability of banding patterns (Table 2). PCR amplifications were carried out in a 20-µL volume containing 20 ng of DNA template, 2.0 µL of 10× reaction buffer (10 mM Tris-HCl, pH9.0, 50 mM KCl, 0.1% TritonX100), 2.5 mM of MgCl2, 1.5 U of Taq DNA polymerase, 0.2 mM of dNTP and 0.2 µM of each single primer. Amplification was performed in an MJ Researcher PTC-200™ programmable thermal controller under the following conditions: heat denaturation at 94°C for 5 min followed by 35 cycles of 30 sec at 94°C, 45 sec at 49°C, 90 sec at 72°C, and a final 7 min extension at 72°C. The amplified fragments were separated by electrophoresis on 2% agarose gels. The gels were stained with ethidium bromide and visualized under UV light and recorded with the aid of a gel documentation system (Gel Doc 1000&2000, Bio-Rad). A 3-kb DNA ladder (MBI Fermentas) was used as a molecular weight marker for comparing amplified fragment size across gels.

**Table 2:** Sequences of primers used for PCR amplification of inter-simple sequence repeat (ISSR), ribosomal internal transcribed spacer (ITS) and chloroplast DNA markers.

ISSR	UBC Primer No.	Nucleotide Sequence^a^
	807	AGA GAG AGA GAG AGA GT
	808	AGA GAG AGA GAG AGA GC
	810	GAG AGA GAG AGA GAG AT
	811	GAG AGA GAG AGA GAG AC
	818	CAC ACA CAC ACA CAC AG
	825	ACA CAC ACA CAC ACA CT
	834	AGA GAG AGA GAG AGA GYT
	835	AGA GAG AGA GAG AGA GYC
	842	GAG AGA GAG AGA GAG AYG
	847	CAC ACA CAC ACA CAC ARC
	866	CTC CTC CTC CTC CTC CTC
	889	DBDACACAC ACA CAC AC
Ribosomal ITS		
		ITS4: TCCTCCGCTTATTGATATGC
		ITS5: GGAAGGAGAAGTCGTAACAAGG
Chloroplast		
	*trn*G-*trn*S	F: GAACGAATCACACTTTTACCAC
		R: GCCGCTTTAGTCCACTCAGC
	*trn*H-*rpl*2	F: CGGATGTAGCCAAGTGGATC
		R: GATAATTTGATTCTTCGTCGCC

a Y: C or T; R: A or G; D: A or G or T; B: C or G or T.

### ISSR data analysis

The amplified ISSR fragments were recorded as presence (1) or absence (0) for each individual. The Ewens-Watterson test for neutrality [20] was performed to examine whether all the ISSR markers used in this study are selectively neutral. Principal coordinate (PCO) analysis was performed on the binary ISSR data matrices using MVSP version 3.13p [21] for each of the geographical locations. Among a variety of different measures of distance or similarity that can be used for PCO analysis, the mean character difference was found to be comparable to several other genetic distance measures for binary data matrices and thus selected for use. The mean character difference distances were measured between the samples directly, and eigen analysis of the distance matrix resulted in direct ordination of the samples. The results were displayed as a two-dimensional scatter plot for visualization of genetic relatedness among individuals at each location.

To test whether *Bruguiera* × *rhynchopetala* is genetically differentiated from parental species to deserve a separate taxonomic status, we examined the clustering pattern among hybrid and other sympatric species, using all 112 individuals in Neighbor-Joining (NJ) analyses. The Jaccard distance matrix, which considers shared presence but not shared absence of ISSR bands as similarity [22], was used to reconstruct the NJ tree in PAUP* 4.0 b [23]. Bootstrap support values were obtained based on 1000 replications.

To evaluate the status of hybrid individuals and examine if there is backcrossing with either parent species or intercrossing among hybrid individuals (i.e., the production of *F*_2_ or later generation), the Bayesian method implemented in NEWHYBRIDS 1.1. [24] was employed. The six genotype classes investigated were: pure parent A, pure parent B, *F*_1_ progeny (50% of the genome originated from parent A and 50% from parent B), *F*_2_ progeny (50% originated from *F*_1_s and 25% from each of the parents A and B), backcrosses with parent A (50% originated from *F*_1_s and 50% from parent A), and backcrosses with parent B (50% originated from *F*_1_s and 50% from parent B; for detail see [25]). Analyses were performed separately for each of the study locations. Each analysis was run independently for three times, starting with a different random number of seeds and for 10^5^ iterations of MCMC chains after 10^4^ burn-in steps, without using any prior information on individual or allele frequency. The affinity of an individual to the respective genotype classes is assessed by posterior probability values.

### Sequence markers

In addition to ISSR, sequences of the nuclear ribosomal internal transcribed spacer (ITS) and two chloroplast intergenic regions (*trn*G-*trn*S, *trn*H-*rpl*2; Table 2) were used for a subset of samples with the aim to examine biparental and maternal relationships among taxa. Results of such were used to infer the direction and extent of introgression. Amplification was performed under the following conditions: heat denaturation at 94°C for 5 min followed by 35 cycles of 30 sec at 94°C, 60 sec at 54 or 55°C, 90 sec at 72°C, and a final 7 min extension at 72°C. The purified amplification products were sequenced directly on an ABI 3100 (Applied Biosystems) automated DNA sequencer with the BigDye terminator cycle sequencing kits. All sequences were deposited in GenBank with the accession numbers presented in Table S1.

All sequences were aligned with ClustalX [26] and manually adjusted with the Sequence Alignment Editor version 1.d1 [27]. For the ITS data, phylogenetic trees were constructed using the maximum likelihood (ML) criterion in RAxML version 7.0.4 [28] and the Bayesian criterion in Mr. Bayes version 3.0b4 [29]. For ML analyses all searches were heuristic with TBR branch swapping. The nucleotide substitution model was first determined by the Akaike Information Criterion (AIC) method with Modeltest version 3.06 [30]. The best-fitting model (GTR+G) and related parameters of the dataset were then used in the ML searches. Bootstrap support (BS) was assessed with 1,000 replicates with the rapid bootstrap algorithm implemented in RAxML [31]. For Bayesian analyses, four Markov chains each initiated with a random tree and with two independent runs each run for 10,000,000 generations, sampling every 100^th^ generation were conducted. Likelihood values were monitored for stationarity with Tracer v1.4.1 [32]. Trees and other sampling points prior to the burn-in cut-off were discarded and the remaining trees were imported into PAUP* v4.0b10 [23] to generate a majority-rule consensus. Posterior probability values [PP; 33] were used to evaluate support of all nodes in the Bayesian trees.

Because the two chloroplast intergenic regions are linked on a haploid genome, sequences were combined and treated as a single marker for analyses. Because gaps were found to be phylogenetically informative among our studied taxa, they were coded as multistate characters with SeqState version 1.32 [34] and appended to the sequence matrices prior to the analyses. The statistical parsimony method of Templeton et al. (1992) [35] implemented in TCS v1.13 [36] was used to construct a haplotype network with the chloroplast data. Compared to phylogenetic trees, this approach appears to be more useful in resolving reticulate relationships [37]. Haplotypes were estimated based on the uncorrected *p*-distances above which the parsimony principle is violated with more than 5% probability. All connections were iteratively joined among haplotypes only when the parsimony has a probability of at least 0.95 of being true as determined by coalescence theory, starting with the shortest distance until all haplotypes are joined or the distance exceeds the parsimony limit [36]. Given chloroplast genome is predominately maternally inherited in flowering plants, the proportion of haplotype sharing between hybrids and parental species reflects the direction of hybridization.

## Results

### Parental origin and genetic relatedness

A total of 284 ISSR marker loci were recorded which represent all fragments amplified with the 12 ISSR primers for 112 individuals belonging to five different taxa (*B. gymnorrhiza*, *B. sexangula*, *B. cylindrica, B*. *parviflora* and the hybrid *B.* × *rhynchopetala*) from four geographically isolated populations. The number of detected fragments differed according to taxa and geographical locations (Table 1 and 3). Genetic differentiation among populations due to geographical isolation resulted in a large number of population-specific bands or alleles within each taxon. Among all the examined taxa, *B.* × *rhynchopetala* was found to contain the highest number of bands despite its relatively small sample size. Over 90% of the bands detected in *B. gymnorrhiza* were present in *B.* × *rhynchopetala*, except for the Johnstone River population where only one hybrid individual was detected based on morphological criteria. This percentage of band sharing was followed by *B. sexangula*, *B. cylindrica*, and *B. parviflora* in descending order (Table 3), though the latter two taxa were found only in the Embley River population. Compared to *B. gymnorrhiza*, fewer bands detected in *B. sexangula* were present in *B.* × *rhynchopetala* (ranging from 56.9–83.9%; Table 3). Only about 50% of the bands detected in *B. parviflora* and *B. cylindrica* (Embley River, Australia) were present in *B.* × *rhynchopetala* (Table 3). When only taxon-specific bands were considered, majority of the bands unique to *B. gymnorrhiza* or *B. sexangula* were observed in *B.* × *rhynchopetala*, whereas *B. parviflora* or *B. cylindrica* bands were rarely present in *B.* × *rhynchopetala*.

**Table 3:** ISSR Band-sharing between Bruguiera × rhynchopetala and sympatric B. gymnorrhiza, B. sexangula, B. cylindrica and B. parviflora.

Population	*B. gymnorrhiza*	*B. sexangula*	*B. cylindrica*	*B. parviflora*
**Australia−**Embley River a				
Total no. (%) of bands shared with *B*. × *rhynchopetala*	103 (91.96%)	83 (77.57%)	55 (52.88%)	32 (45.71%)
No. of species-specific bands	25	26	36	24
No. (%) of species-specific bands found in *B*. × *rhynchopetala*	21 (84%)	15 (57.69%)	4 (11.11%)	1 (4.17%)
**Australia−**Johnstone River				
Total no. (%) of bands shared with *B*. × *rhynchopetala*	78 (62.90%)	57 (72.15%)		
No. of species-specific bands	70	22		
No. (%) of species-specific bands found in *B*. × *rhynchopetala*	26 (37.14%)	5 (22.73%)		
**Hainan**				
Total no. (%) of bands shared with *B*. × *rhynchopetala*	145 (92.36%)	109 (83.85%)		
No. of species-specific bands	58	35		
No. (%) of species-specific bands found in *B*. × *rhynchopetala*	52 (89.66%)	15 (42.86%)		
**Indonesia**				
Total no. (%) of bands shared with *B*. × *rhynchopetala*	117 (93.6%)	62 (56.88%)		
No. of species-specific bands	66	42		
No. (%) of species-specific bands found in *B*. × *rhynchopetala*	58 (87.88%)	5 (11.90%)		

a Australia**-**Embley River is the only sample site where four species, *B. gymnorrhiza*, *B. sexangula*, *B. cylindrica* and *B. parviflora*, occur in sympatry.

**Figure 1:**
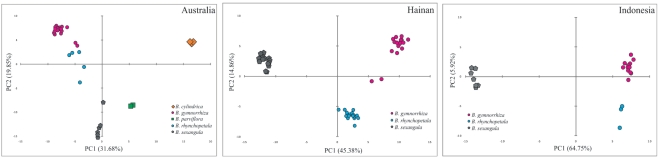
Two-dimensional scatter plot obtained from principal coordinate analysis of ISSR data for *Bruguiera* individuals sampled from (A) Australia (including both sites ER and JR), (B) Hainan, and (C) Indonesia. Symbols of respective taxa are indicated below graph and locality information is given in Table 1.

The four *Bruguiera* species, *B. gymnorrhiza, B. sexangula, B. parviflora,* and *B. cylindrica*, were clearly separated along the first two axes in the scatter plots (Figure 1), consistent with their taxonomic identification. By contrast, *B.* × *rhynchopetala* could be demarcated at some but not all studied sites, albeit with varying degree of intermediacy between *B. gymnorrhiza* and *B. sexangula*. For example, in Australia (sites ER and JR), two of the five hybrid individuals were positioned intermediate between *B. gymnorrhiza* and *B. sexangula*, but the other three hybrids were all positioned closely to *B. gymnorrhiza* (Figure 1A). On the other hand, hybrids sampled from Hainan Island were more clustered with one another and were all positioned more or less intermediate between *B. gymnorrhiza* and *B. sexangula* along axis 1 (Figure 1B). In contrast, the three hybrid individuals from Indonesia were all positioned closely to *B. gymnorrhiza* (Figure 1C). The proportion of band sharing (Table 3) and genetic relatedness revealed in the scatter plots (Figure 1) support the morphological hypothesis that *B. gymnorrhiza* and *B. sexangula* are the parental species of *B.* × *rhynchopetala*. The varying degree of relatedness between the hybrids and the two parental species suggests that introgression occurs within populations and that such introgression is mostly unidirectional, i.e., the hybrid may preferentially if not exclusively backcrosses to one parent only.

### Direction of hybridization and introgression

**Figure 2:**
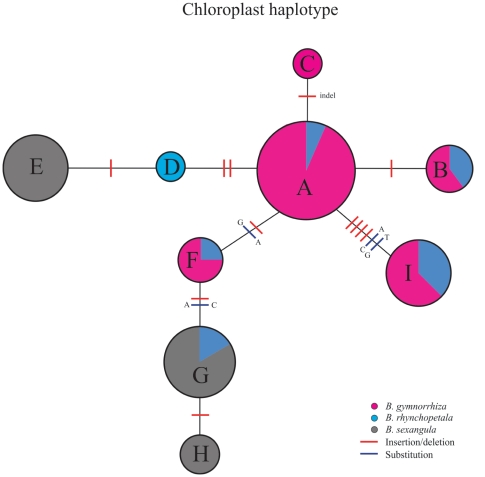
Statistical parsimony network of haplotypes A-I (see **Table 4**) obtained from combined chloroplast sequences of *B. gymnorrhiza*, *B. sexangula*, and *B*. × *rhynchopetala* individuals. Sizes of circles are approximately proportional to the number of individuals with the given haplotype. Bars on lines between circles represent site changes between haplotypes under the statistical parsimony criterion.

A total of nine haplotypes labelled as A-I (Table 4) were detected based on the chloroplast data and they were distinguished from each other by 1–6 mutations in the haplotype network (Figure 2). Five haplotypes (A, B, C, F, and I) belonged to *B. gymnorrhiza* and four of these haplotypes were shared with *B.* × *rhynchopetala.* In comparison, only one out of the three haplotypes of *B. sexangula* (E, G, and H) was found in *B.* × *rhynchopetala*. Haplotype D appears to be unique to *B.* × *rhynchopetala* which is not found in either of the parental taxa. This hybrid-specific haplotype could be a product of intercrossing among hybrids and fast evolutionary rate at a microsatellite site, or due to insufficient sampling of parental taxa. The chloroplast data indicate mother-hybrid relationships of *B.* × *rhynchopetala* with both *B. gymnorrhiza* and *B. sexangula*. However, the asymmetrical pattern of haplotype sharing suggests a predominant maternal role of *B. gymnorrhiza* during hybridization.

**Table 4:** Summary of chloroplast haplotypes detected in *B. gymnorrhiza*, *B. sexangula*, and *B*. × *rhynchopetala* (indicated in bold).

Haplotype	Individuals
A	BG1135In, BG1144In, BG1148In ( = BG5In/BG5HN), BG1161In, BG390ER, BG391ER, BG401ER, BG407ER, BG408ER, BG409ER, BG412ER, BG414ER, BG421ER, BG453ER, **BR1160In**
B	BG1142In, BG1143In, BG1150In, **BR1134In**, **BR1166In**
C	BG1003JR, BG1093JR
D	**BR404ER, BR1065JR**
E	BS389ER, BS392ER, BS397ER, BS400ER, BS402ER, BS403ER, BS1066JR, BS1090JR^a^, BG420ER^b^
F	BG6HN, BG18HN, BG20HN, **BR20HN**
G	BS9HN, BS11HN, BS20HN, BS1153In, BS1154In ( = BS4HN), **BR9HN**
H	BS1151In, BS1152In, BS1159In
I	BG405ER, BG411ER, BG422ER, BG423ER, BG428ER, **BR381ER**, **BR454ER, BR455ER**

The number of individuals included from each site and locality label can be found in Table 1.

GenBank accession numbers are provided in Table S1.

**Figure 3:**
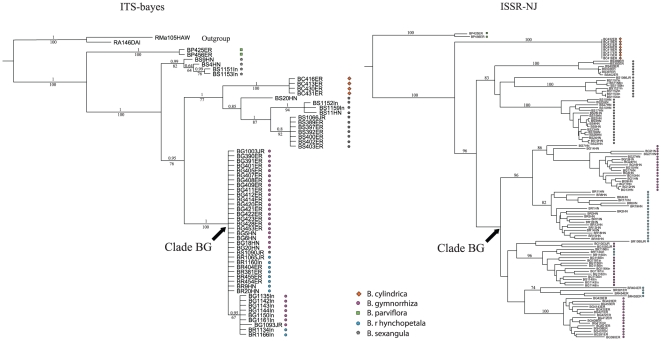
Phylogenetic relationships of *B.* × *rhynchopetala* with *B. gymnorrhiza, B. sexangula*, *B. cylindrica*, and *B. parviflora* based on nuclear genomic data. (**A**) Bayesian tree based on ribosomal ITS data using the GTR+gamma model (base frequencies A = 0.21, C = 0.36, T = 0.13, and G = 0.30; and gamma = 0.26). Bootstrap (BS; above branch; based on ML analyses) and posterior probability (PP; below branch) values >50% are indicated. Individuals of *Rhizophora* were used as outgroup. (**B**) Neighbor-joining tree of 112 individuals from all sample sites based on Jaccard distances calculated from the ISSR markers. Numbers above clades are bootstrap support values (values below 50% are not given).

Statistical parsimony network of these haplotypes is shown in Figure 2.

a BS1090JR is likely a hybrid, which has the same haplotype E as some of the individuals of *B. sexangula*, but is grouped with *B. gymnorrhiza* and *B*. × *rhynchopetala* based on nuclear markers (see Figure 3); ^b^BG420ER is also likely a hybrid which has the same haplotype E as some of the individuals of *B. sexangula*, but is grouped with individuals of *B. gymnorrhiza* and *B*. × *rhynchopetala* from the same site based on nuclear markers.

Apart from the chloroplast data, nuclear data also reveal a similar pattern of genetic association between *B.* × *rhynchopetala* and *B. gymnorrhiza*. All hybrid individuals were found to be nested in the clade that contains exclusively *B. gymnorrhiza* in both the ITS and ISSR trees (namely clade BG; Figure 3). Surprisingly, none of the morphological hybrids were found to be closely related to *B. sexangula.* The clade BG is shown to be sister to *B. sexangula* and *B. cylindrica* regardless of geographical localities. Among all, *B. parviflora* is clearly genetically distant from the rest of the taxa. These relationships appear to be a result of unidirectional backcrossing of hybrids to *B. gymnorrhiza*, which leads to significant gene introgression.

### Hybrid status

**Figure 4:**
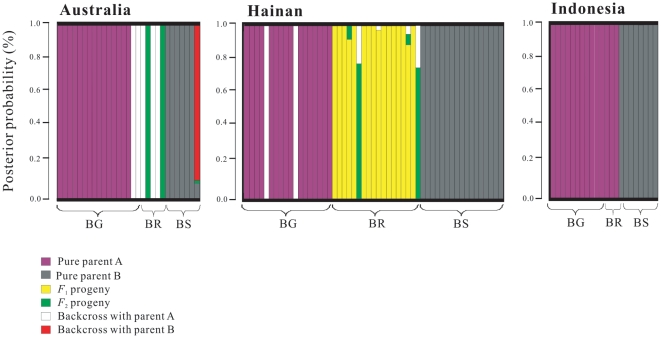
Bayesian inference of genotype class estimated with NewHybrids among individuals of *B.* × *rhynchopetala*, *B. gymnorrhiza,* and *B. sexangula* based on ISSR data. (**A**) Australia, including both sites ER and JR, (**B**) Hainan, and (**C**) Indonesia. The genotype classes are represented by colors, and individuals are represented as columns. Within each column (individual) the extent of the component colors indicates the posterior probability of an individual with respect to each genotype class. BG: *B. gymnorrhiza*; BS: *B. sexangula*; BR: *B*. × *rhynchopetala.*

NewHybrids analyses indicated that individuals of *B.* × *rhynchopetala* are a mix of primarily *F*_1_s and backcross progeny with *B. gymnorrhiza*, with rare presence of *F*_2_s and backcross progeny with *B. sexangula* (Figure 4). For example, hybrids from Hainan are mainly *F*_1_s, but *B.* × *rhynchopetala* from Australia (sites ER and JR) contains a mixture of backcrosses and likely *F*_2_s. While *B.* × *rhynchopetala* from the Indonesia population are considered as hybrids based on morphological features, NewHybrids analyses did not support this interpretation but indicated that these individuals may belong to *B. gymnorrhiza* (Figure 4). However, re-amplifications with ISSR primer 818 confirmed the presence of *B. sexangula-*specific bands in the Indonesian hybrids. Given only six genotype classes were specified in the NewHybrids analyses, it is likely that these hybrids represent progeny after several generations of backcrossing (i.e., introgressants of advanced generations) that could no longer be detected as hybrids based on the molecular data.

## Discussion

Recent advances in molecular technology have offered unprecedented opportunities for fine analysis of natural hybridization at genetic and genomic scale in plants [10], [12]. Of the various genetic approaches to studying hybridization in plants, inter-simple sequence repeat (ISSR) is one of the simplest molecular methods that can be used to generate a large number of molecular markers across the genome for comparative analysis of the putative hybrids and their parental species on a broad geographical scale, as demonstrated in the present study. In addition, chloroplast and nuclear sequence markers provide an alternative means in elucidating the direction of hybridization and introgression at the species level. A combination of these molecular methods shed light on the origin and evolution of the mangrove hybrid *B.* × *rhynchopetala*, which was previously unclear based on morphological characters alone.

### Detection of hybridization

Many studies that use molecular markers to test hybridization hypotheses rely on the expectation that hybrids show additive marker profiles; and either all, or nearly all, parent-diagnostic markers should be found in a hybrid [11], [38]. For example, the hybrid status of *Helianthus paradoxus* is supported by its possession of a combination of allozyme and rDNA alleles found in *H. annuus* and *H. petiolaris* [39]. The extent of polymorphism in the parental species also affects the expectation of marker additivity [40]. Complete or nearly complete additivity in hybrids is expected only if heterozygosity and polymorphism are low within parental species and/or hybrid segregation or recombination is negligible (e.g., in *F*_1_ generation) [38], [41].

In the present study, some of the ISSR primers revealed apparent band additivity in *B.* × *rhynchopetala* from Hainan Island (e.g., Figure S1), but the band-sharing statistics computed over all primers are more informative on genomic additivity of the hybrid in other populations. Based on the combined ISSR data of all marker loci, the hypothetical hybridization between *B. gymnorrhiza* and *B. sexangula* can be confirmed. Interspecific polymorphism at marker loci provided a large number of species-specific bands for differentiating sympatric *Bruguiera* species. Most of the *B. gymnorrhiza*- and *B. sexangula*-specific bands were present in the hybrid genomic profile, supporting the hypothesis that they were the parental species involved in the hybridization events. Compared to ITS and cpDNA sequencing analyses, the scatter plots from PCO analysis can most effectively separate *B.* × *rhynchopetala* from its parental species.

The possible involvement of sympatric *B. cylindrica* or *B. parviflora* in hybridization can be eliminated as their species-specific bands were much rarer in the hybrids. However, it is noteworthy that not all ISSR bands as well as chloroplast haplotypes present in *B. × rhynchopetala* were found in their respective parental populations (Table 3 and 4). The few unique genetic features of *B. × rhynchopetala* could be due to polymorphism within each parental population (the percentage of polymorphic loci ranging from 37.50–50.32% in *B. gymnorrhiza* and 16.51–24.62% in *B. sexangula*). Relative to the large number of ISSR loci surveyed, the samples included in this study could not possibly contain all existing alleles within the natural population of the respective parental species. Furthermore, given that some hybrid individuals could be *F*_2_ or introgressants of advanced generations (Figure 4), these *B. × rhynchopetala*-specific ISSR fragments and chloroplast haplotype could be a result of recurrent intercrossing or new mutations in the hybrids. Another explanation for those missing parental bands in *B. × rhynchopetala* could be due to the dominant nature of ISSR markers. When hybrids are screened for the presence or absence of a parental marker, it may not be found if the parental genotype was heterozygous for the dominant marker [38]. While co-dominant markers such as microsatellites are known to offer major advantages over dominant markers for revealing Mendelian genotypes and in discriminating hybrids in some cases [e.g., 42–44], the usage of microsatellites appears to be limited to only closely-related hybridizing species. Primers designed in one species may not be widely applicable across the genus when determining parental origins of a hybrid taxon.

### Evidence of introgression

The variation in scatterness among hybrids in the PCO plots (Figure 1) and results of the NewHybrids analyses (Figure 4) clearly indicate that *B.* × *rhynchopetala* consists of *F*_1_s, *F*_2_s and introgrexssants. Although the hybrid samples from Hainan population are all well separated from the parental species and most of them are *F*_1_s (Figure 1, S1 and 4), *F*_2_s or progeny of backcrosses with *B. gymnorrhiza* apparently exist in this population (Figure 4), resulting in their closer genetic affinity to *B. gymnorrhiza* than to *B. sexangula* in the nuclear data (Figure 3). Similarly, two of the five morphological hybrids in the two Australian populations are likely *F*_2_s and the rest are apparently progeny of backcrosses with *B. gymnorrhiza* (Figure 4). In the case of the Indonesian population, although the three hybrid samples exhibit intermediate morphological characters between *B. gymnorrhiza* and *B. sexangula*, they are genetically much closer to *B. gymnorrhiza* than to *B. sexangula* (Figure 1 and 3). These hybrids could belong to more advanced generations of introgressants, which cannot be accurately detected in the NewHybrids analysis (Figure 4).

If *B.* × *rhynchopetala* consisted of only *F*_1_s, the species-specific bands from *B. gymnorrhiza* and *B. sexangula* should have about equal presence in the hybrid genome. However, a pattern emerges that more *B. gymnorrhiza-*specific bands were present in *B.* × *rhynchopetala*, exceeding the percentage of *B. sexangula-*specific bands by 26–76% in our studied populations regardless of sample size (Table 3). This pattern of nonsymmetrical band-sharing provides strong evidence for a unidirectional introgression between the hybrid and *B. gymnorrhiza* in at least three of the four mangrove forests. A higher level of pollen dispersal from *B. gymnorrhiza* or a larger *B. gymnorrhiza* population size compared to *B. sexangula* could result in unidirectional introgression. Our chloroplast DNA data suggest that both *B. gymnorrhiza* and *B. sexangula* can serve as the pollen recipient (i.e., maternal parent) when the two species coexist and hybridize (Figure 2). Although a field survey showed that the number of mature individuals of *B. sexangula* is about twice that of *B. gymnorrhiza* in the hybrid zone in Hainan, the majority of *B*. × *rhynchopetala* samples had the *B. sexangula* chloroplast genotype [45], indicating that *B. gymnorrhiza* acted primarily as a pollen donor in the hybridization and introgression events. In addition to asymmetrical pollination from parental species, other pre- or post-mating isolation mechanisms may exist between the hybrids and *B. sexangula*, which could effectively prevent backcrosses to *B. sexangula* in some of the populations. However, the direction of introgression may vary among geographical locations. For example, a sample from site JR of Australia was morphologically classified as *B. sexangula* but genetically identified as most likely an introgressant (Figure 4).

### Mechanisms of reproductive isolation

Natural hybridization plays an important role in plant speciation and evolution. Knowledge of the extent of hybridization and introgression is relevant in predicting evolutionary fate of the hybrids as well as the parental species. In theory, introgressive hybridization can prevent genetic differentiation necessary for hybrid speciation, and at the same time, result in continuous gene flow from one species into the other and hence blur the previously established species boundaries. One question arises from this evolutionary dynamic – how could sympatric *Bruguiera* taxa remain as distinct species if hybridization and introgression occur in all geographical regions?

Different flower sizes and pollination mechanisms have been reported for different *Bruguiera* species. The two large-flowered *B. gymnorrhiza* and *B. sexangula* are pollinated mostly by birds, and the two small-flowered species *B. cylindrica* and *B. parviflora* are pollinated mainly by insects [1]. These major differences can lead to prezygotic reproductive isolation between the two groups. Within the small-flowered species group, no intermediate morphs were found between sympatric *B. cylindrica* and *B. parviflora*. Within the large-flowered species group, similarities in floral morphology, phenology, and pollination mechanisms all facilitate hybridization. Lack of pre- or post-mating isolation mechanisms and potentially a high degree of genome compatibility between sympatric *B. gymnorrhiza* and *B. sexangula* have resulted in fertile hybrids in sites of sympatry. However, the rarity of hybrids in some of the sympatric populations, such as in Indonesia and Australia, implies that hybridization between the two large-flowered species may not be as frequent as their extensive sympatry suggests. This could be due to a shorter overlapping flowering period between the two species in these locations. The timing and duration of flowering are affected by climate and other ecological conditions which vary according to geographical locations. In northeastern Australia, *B. gymnorrhiza* and *B. sexangula* overlap in flowering time only in August and the hybrid flowers in August-September [46]. In contrast, favorable environmental conditions in Hainan permit nearly year-long flowering in both species, and hence provide more opportunities for hybridization. Moreover, the hybrids in Hainan also have a relatively long flowering time from March-June, providing ample time for introgression. Although interspecific gene flow through frequent hybridization could potentially lead to morphological convergence between *B. gymnorrhiza* and *B. sexangula*, at least in Hainan, the two species can still be separated genetically in accordance with their taxonomic identifications. This indicates that introgressive hybridization in *Bruguiera* has not yet resulted in convergent evolution.

Several intermediate forms of putative hybrid origin also exist in a closely related mangrove genus, *Rhizophora*, including *Rhizophora lamarckii* Montr. or *Rhizophora* × *lamarckii* [1], *Rhizophora* × *annamalayana* [47] and *Rhizophora* × *selala* [48]. Recent molecular studies have confirmed that *R.* × *lamarckii* is a hybrid between *Rhizophora apiculata* and *Rhizophora stylosa*; *R*. × *annamalayana* is a hybrid between *R. apiculata* and *Rhizophora mucronata*; and *R. selala* is a hybrid between *R. stylosa* and *Rhizophora samoensis* in the Indo-West Pacific region [49]. Despite frequent hybridization in sympatric sites, reproductive isolation between all the parental species of *Rhizophora* is ensured by *F*_1_ hybrid sterility. However, this is not the case in *Bruguiera*, as no hybrid sterility or reduced fertility is observed for *B.* × *rhynchopetala*. As shown in this study, *F*_2_s and introgressants exist within the hybrid populations, though the frequency of hybridization and extent of introgression apparently vary among geographical locations differing in climatic and ecological conditions.

### The effects of sample size

As mentioned earlier, the difference in sample sizes among sites in the present study is primarily due to difference in the frequency of hybrids that could be morphologically identified at the time of field collection. The extensive overlapping in flowering phenology in Hainan apparently facilitates hybridization between *B. gymnorrhiza* and *B. sexangula*, resulting in a high frequency of *F*_1_ hybrids and thus a discernible hybrid zone. In contrast, *F*_1_ hybrids are rare in the Indonesian and Australian populations and the introgressants are difficult to detect in the field. Consequently, natural hybridization between *Bruguiera* species has often gone undetected in most of Indo-West Pacific region where environmental conditions and flowering phenology significantly differ from those existing in Hainan. Thus our results provide the most needed genetic evidence showing that introgressive hybridization between *B. gymnorrhiza* and *B. sexangula* actually occurs over a wide range of geographical locations.

To further examine whether the difference in sample sizes has any significant impact on the results of this study, we compared the two Australian sites, ER and JR. Despite their large differences in the sample sizes of parents and morphologically identifiable hybrids, the same results and conclusions can be reached based on our genetic analyses. Even though the sample size may affect the total number of ISSR fragments detected for each taxon within each population, it does not affect the detectability of hybrids in this study. For example, only one hybrid (field identification number 1065) from site JR could be ascertained based on morphological criteria during field sampling and our genetic analysis recognized it to be an *F*_2_. On the other hand, sample ID 1090 from the same site, which was uncertainly assigned to *B. sexangula* based on its morphology, is likely to be a hybrid as indicated by its conflicting affiliations with the two parental species between nuclear and cpDNA analyses. Similarly, all morphologically identifiable hybrids from site ER were confirmed by our genetic analyses. In addition, two other samples assigned as *B. gymnorrhiza* in the field are shown to be introgressants using the combined nuclear and cpDNA analyses. Because some backcross hybrids, especially those introgressants of advanced generations, resemble either parent in the diagnostic morphological characteristics, they could not be recognized as hybrids during field collection. Therefore more introgressants might be detected genetically by increasing sample sizes of the putative parents. However, the results will only lend further support to our conclusions in the present study.

## Conclusions

In the Indo-West Pacific region, *Bruguiera × rhynchopetala* was shown to comprise *F*_1_s, *F*_2_s and different generations of introgressants. The extent of hybridization and introgression varies among *Bruguiera* populations according to their geographical locations. Among various analyses, the PCO scatter plots and NewHybrids analysis based on ISSR data can most effectively distinguish the hybrid from its parental species. However, genetic affinities shown in both ISSR and ITS phylogenies indicate that *B.* × *rhynchopetala* has not sufficiently differentiated from *B. gymnorrhiza* to deserve a distinct species status. In addition, ISSR data provide strong support for multiple independent origins of *B.* × *rhynchopetala*, as the hybrid individuals from different geographical locations form separate genetic clusters. These hybrids occur only within the parental habitats, and there is no observable ecological differentiation from the parents other than a shorter flowering period. Thus, we conclude that lack of reproductive isolation between *B.* × *rhynchopetala* and its parental species has resulted in introgression, and the persistence of *B.* × *rhynchopetala* can be accounted for by recurrent hybridization between sympatric *B. gymnorrhiza and B. sexangula* in the Indo-West Pacific region.

## Supporting Information

Figure S1 ISSR fragments amplified by one of the 12 primers (UBC Primer No. 818; see Table 2), showing band-sharing between individuals of *Bruguiera × rhynchopetala* (lanes 12–21) and sympatric *B. gymnorrhiza* (lanes 2–11) and *B. sexangula* (lanes 22–30) in Hainan. Interspecific polymorphism at marker loci provided species-specific bands for differentiating sympatric *Bruguiera* taxa. Most of the *B. gymnorrhiza-* and *B. sexangula*-specific bands were present in the hybrid genomic profile. M: DNA ladder (lane 1) was used as a molecular weight marker for comparing amplified fragment size across gels. Arrows mark the first sample of each taxon.
(EPS)

Table S1 GenBank accession number of ribosomal ITS and chloroplast DNA regions from *Bruguiera* individuals used in this study. BC: *B. cylindrica*; BG: *B. gymnorrhiza*; BP: *B. parviflora*; BR: *B*. × *rhynchopetala*; BS: *B. sexangula*. Locality label can be found in Table 1.
(DOC)
