# Supplementary material for: Genomic Markers Reveal Introgressive Hybridization in the Indo-West Pacific Mangroves: A Case Study
Source: PLoS One. 2011 May 11;6(5):e19671. doi: 10.1371/journal.pone.0019671 (PMC3092761; doi:10.1371/journal.pone.0019671)
Supplement: Table S1 — GenBank accession number of ribosomal ITS and chloroplast DNA regions from Bruguiera individuals used in this study. BC: B. cylindrica; BG: B. gymnorrhiza; BP: B. parviflora; BR: B. × rhynchopetala; BS: B. sexangula. Locality label can be found in Table 1. (DOC) [file pone.0019671.s002.doc]

**Table S1.** GenBank accession number of *Bruguiera* individuals for the ribosomal ITS and chloroplast regions used in this study.

| Sample ID^a^ | ITS | *trn*H-*rpl*2 | *trn*G-*trn*S |
| --- | --- | --- | --- |
| BC413ER | HM366078 | HM365958 | HM366018 |
| BC416ER | HM366079 | HM365959 | HM366019 |
| BC430ER | HM366080 | HM365960 | HM366020 |
| BC431ER | HM366081 | HM365961 | HM366021 |
| BG5In | HM366082 | HM365962 | HM366022 |
| BG6HN | HM366083 | HM365963 | HM366023 |
| BG18HN | HM366084 | HM365964 | HM366024 |
| BG20HN | HM366085 | HM365965 | HM366025 |
| BG390ER | HM366086 | HM365966 | HM366026 |
| BG391ER | HM366087 | HM365967 | HM366027 |
| BG401ER | HM366088 | HM365968 | HM366028 |
| BG405ER | HM366089 | HM365969 | HM366029 |
| BG407ER | HM366090 | HM365970 | HM366030 |
| BG408ER | HM366091 | HM365971 | HM366031 |
| BG409ER | HM366092 | HM365972 | HM366032 |
| BG411ER | HM366093 | HM365973 | HM366033 |
| BG412ER | HM366094 | HM365974 | HM366034 |
| BG414ER | HM366095 | HM365975 | HM366035 |
| BG420ER | HM366096 | HM365976 | HM366036 |
| BG421ER | HM366097 | HM365977 | HM366037 |
| BG422ER | HM366098 | HM365978 | HM366038 |
| BG423ER | HM366099 | HM365979 | HM366039 |
| BG428ER | HM366100 | HM365980 | HM366040 |
| BG453ER | HM366101 | HM365981 | HM366041 |
| BG1003JR | HM366102 | HM365982 | HM366042 |
| BG1093JR | HM366103 | HM365983 | HM366043 |
| BG1135In | HM366104 | HM365984 | HM366044 |
| BG1142In | HM366105 | HM365985 | HM366045 |
| BG1143In | HM366106 | HM365986 | HM366046 |
| BG1144In | HM366107 | HM365987 | HM366047 |
| BG1150In | HM366108 | HM365988 | HM366048 |
| BG1161In | HM366109 | HM365989 | HM366049 |
| BP425ER | HM366110 | HM365990 | HM366050 |
| BP456ER | HM366111 | HM365991 | HM366051 |
| BR9HN | HM366112 | HM365992 | HM366052 |
| BR20HN | HM366113 | HM365993 | HM366053 |
| BR381ER | HM366114 | HM365994 | HM366054 |
| BR404ER | HM366115 | HM365995 | HM366055 |
| BR454ER | HM366116 | HM365996 | HM366056 |
| BR455ER | HM366117 | HM365997 | HM366057 |
| BR1065JR | HM366118 | HM365998 | HM366058 |
| BR1134In | HM366119 | HM365999 | HM366059 |
| BR1160In | HM366120 | HM366000 | HM366060 |
| BR1166In | HM366121 | HM366001 | HM366061 |
| BS4HN | HM366122 | HM366002 | HM366062 |
| BS9HN | HM366123 | HM366003 | HM366063 |
| BS11HN | HM366124 | HM366004 | HM366064 |
| BS20HN | HM366125 | HM366005 | HM366065 |
| BS389ER | HM366126 | HM366006 | HM366066 |
| BS392ER | HM366127 | HM366007 | HM366067 |
| BS397ER | HM366128 | HM366008 | HM366068 |
| BS400ER | HM366129 | HM366009 | HM366069 |
| BS402ER | HM366130 | HM366010 | HM366070 |
| BS403ER | HM366131 | HM366011 | HM366071 |
| BS1066JR | HM366132 | HM366012 | HM366072 |
| BS1090JR | HM366133 | HM366013 | HM366073 |
| BS1151In | HM366134 | HM366014 | HM366074 |
| BS1152In | HM366135 | HM366015 | HM366075 |
| BS1153In | HM366136 | HM366016 | HM366076 |
| BS1159In | HM366137 | HM366017 | HM366077 |

^a^ BC: *B. cylindrica*; BG: *B. gymnorrhiza*; BP: *B. parviflora*; BR: *B*. × *rhynchopetala*. Locality label ER, HN, IN, and JR can be found in Table 1. BG5In=BG1148In; BS4HN=BS1154In.
